# Analysis of copy number variations and possible candidate genes in spontaneous abortion by copy number variation sequencing

**DOI:** 10.3389/fendo.2023.1218793

**Published:** 2023-10-17

**Authors:** Wei Bai, Qi Zhang, Zhi Lin, Jin Ye, Xiaoqi Shen, Linshuang Zhou, Wenpin Cai

**Affiliations:** ^1^ Department of Laboratory Medicine, Wenzhou Traditional Chinese Medicine Hospital of Zhejiang Chinese Medical University, Zhejiang, China; ^2^ Key Laboratory of Digital Technology in Medical Diagnostics of Zhejiang Province, Dian Diagnostics Group Co., Ltd., Hangzhou, China

**Keywords:** copy number variation, miscarriage, maternal age, gestational age, body mass index (BMI), copy number variation sequencing (CNV-seq)

## Abstract

**Introduction:**

Embryonic chromosomal abnormalities represent a major causative factor in early pregnancy loss, highlighting the importance of understanding their role in spontaneous abortion. This study investigates the potential correlation between chromosomal abnormalities and spontaneous abortion using copy number variation sequencing (CNV-seq), a Next-Generation Sequencing (NGS) technology.

**Methods:**

We analyzed Copy Number Variations (CNVs) in 395 aborted fetal specimens from spontaneous abortion patients by CNV-seq. And collected correlated data, including maternal age, gestational week, and Body Mass Index (BMI), and analyzed their relationship with the CNVs.

**Results:**

Out of the 395 cases, 67.09% of the fetuses had chromosomal abnormalities, including numerical abnormalities, structural abnormalities, and mosaicisms. Maternal age was found to be an important risk factor for fetal chromosomal abnormalities, with the proportion of autosomal trisomy in abnormal karyotypes increasing with maternal age, while polyploidy decreased. The proportion of abnormal karyotypes with mosaic decreased as gestational age increased, while the frequency of polyploidy and sex chromosome monosomy increased. Gene enrichment analysis identified potential miscarriage candidate genes and functions, as well as pathogenic genes and pathways associated with unexplained miscarriage among women aged below or over 35 years old. Based on our study, it can be inferred that there is an association between BMI values and the risk of recurrent miscarriage caused by chromosomal abnormalities.

**Discussion:**

Overall, these findings provide important insights into the understanding of spontaneous abortion and have implications for the development of personalized interventions for patients with abnormal karyotypes.

## 1 Introduction

Approximately 12%-15% of all clinically recognized pregnancies end in loss (1, 2), and fetal chromosomal abnormalities are one of the most common causes (3). Fetal chromosomal abnormalities include chromosome number abnormalities and chromosome structure abnormalities. Numerical abnormalities refer to errors in the number of chromosomes, which can be classified into two main categories: aneuploidy and polyploidy (4). Aneuploidy involves the gain or loss of one or more chromosomes. The most common form of aneuploidy is trisomy, which involves three copies of the particular chromosome instead of the normal two. Monosomy is another form of aneuploidy, that only one copy of a particular chromosome. In addition to trisomy and monosomy, other types of numerical abnormalities can occur, such as tetrasomy (four copies of a particular chromosome) and nullisomy (no copies of a particular chromosome)(5). These abnormalities are mainly caused by non-disjunction during cell division or other mechanisms (6–9). Polyploidy refers to the presence of one or more extra sets of chromosomes. It is mainly caused by meiotic errors and abnormal fertilization (6, 7). Structural abnormalities refer to changes in the structure of chromosomes, such as deletions, duplications, inversions, or translocations (10, 11). Chromosomal structural abnormalities can be caused by DNA replication errors, or breakage and rearrangement of chromosomes due to various internal and external factors, such as exposure to radiation, chemicals, viruses, or genetic factors (7, 12). So, these types of abnormalities can occur spontaneously or be inherited from a parent (13–16). Chromosomal mosaicism refers to the existence of two or more different cell populations in the body. It can be caused by errors occurring during gamete meiosis. For instance, after the formation of trisomies, a fraction of cells may undergo trisomy rescue, leading to the development of mosaic embryos. Additionally, early embryonic development mitotic errors can also contribute to emergence of mosaicism (17–19).

In addition, CNVs can be further categorized into *de novo* and inherited variants. *De novo* CNVs refer to genomic alterations that arise spontaneously during embryonic or fetal development. These variations are not inherited from the parental genomes and occur as novel changes in the DNA sequence. On the other hand, inherited CNVs are genetic changes passed down from one or both parents to the offspring. These variants can be present in the parental genomes and transmitted through generations.

Though the exact mechanism of how chromosomal abnormalities lead to miscarriage is not yet fully understood, but it is believed to occur due to embryo implant or development failure. In certain scenarios, chromosome abnormalities cause miscarriage by disrupting normal embryonic development or leading to abnormal implantation in the uterus (20, 21). Alternatively, the abnormalities may cause early fetal death or developmental defects that are incompatible with life (7, 22, 23). In summary, understanding chromosomal abnormalities in miscarriage by fetal tissue testing is essential for identifying potential causes and providing appropriate counseling for future pregnancies.

With the development of molecular biology detection technology, the methods for fetal tissue testing are constantly updated. The traditional karyotyping technique is the gold standard for detecting chromosomal abnormalities, but it is complex, time-consuming, and carries a high risk of failure. Furthermore, this technique is not suitable for analyzing CNVs smaller than 5 Mb (24). Compared to that, Fluorescent *in situ* hybridization (FISH) has solved the problem of cell culture and shortened the operation time, but it cannot fully reflect the chromosome situation of abortion tissues limited by the number of specific fluorescent probes (24–26). Chromosomal microarray analysis (CMA) is advanced with a high detection success rate, high resolution, and short detection period (24, 27). However, it is not widely used because of its high price. In recent years, with the widespread application of next-generation sequencing (NGS) technology, more research institutions have developed chromosome abnormality detection techniques based on the NGS platform. In 2009, Xie et al. (28) first reported copy number variation sequencing (CNV-seq), which can detect chromosomal abnormalities based on NGS. CNV-seq is a low-depth detection technique that can detect and quantify chromosomal mosaicism with high sensitivity and specificity. CNV-seq has a simpler protocol, faster diagnostic turnaround time, and relies on genome-wide uniformly distributed reads mapped to sequential bins across all chromosomes, which avoids the bias and noise introduced by probes or amplification (19, 28, 29).

Additionally, CNV-seq has played a pivotal role in enabling comprehensive profiling of CNVs and identifying potential candidate genes associated with adverse pregnancy outcomes, Wu et al. (30) utilized CNV-seq to analyze 505 fetal samples and found a significant correlation between the occurrence of chromosomal abnormalities and gestational age. This study identified 168 genes in the uncertain significance (VUS) CNVs and pathogenic CNVs (pCNVs) region. Enrichment analysis revealed that these genes are involved in multiple functions and pathways associated with embryonic development. Similarly, Zhang et al. (31) analyzed 695 fetal samples using CNV-seq and observed different trends in the occurrence of numerical and structural chromosomal abnormalities with increasing age and gestational age. Furthermore, their GO enrichment analysis revealed enrichments of 42 functions primarily associated with brain and nervous system development, heart formation and development, and organ formation. Moreover, Yi et al. (32) investigated the genetic etiology distribution of 398 fetuses with congenital heart disease in the prenatal setting using CNV-seq and whole exome sequencing (WES). They identified chromosomal abnormalities in 97 cases (24.37%), including 43 aneuploids, 53 pCNVs, and 1 case with both aneuploids and pCNVs.

In our study, we utilized CNV-seq to identify chromosomal abnormalities in tissue samples obtained from individuals who experienced spontaneous abortion. We also collected data on maternal age, gestational week, and Body Mass Index (BMI) to assess potential correlations with chromosomal abnormality patterns. Furthermore, by conducting gene enrichment analysis, we aimed to uncover potential candidate genes and pathways associated with miscarriage in different age groups. This study enhanced our understanding of the genetic landscape of chromosomal abnormalities and their impact on adverse pregnancy outcomes.

## 2 Materials and methods

### 2.1 Participants

We collected a total of 395 specimens from pregnant women who had experienced spontaneous abortions during the period between March 2018 and June 2022 and were admitted to the Wenzhou Hospital of Traditional Chinese Medicine Affiliated Zhejiang University of Traditional Chinese Medicine. All participants had experienced either sporadic or recurrent miscarriages, and voluntarily underwent CNV-seq testing following the loss of their pregnancies. Additionally, they provided signed informed consent before participating in the study. The age range of the participants was between 21 and 45 years. We divided the pregnant women into two groups: those under 35 years old who were in the non-advanced age group, and those who were aged 35 years or older, in the advanced age group. Their gestational age ranged from 3 to 23 weeks.

### 2.2 Statistical analysis

We used the statistical software SPSS 26.0 to conduct chi-square tests (for expected values greater than 5 and n > 40) and Fisher’s exact tests (for expected values less than 5 and n < 40) on count data. A *p*-value < 0.05 was considered statistically significant. Furthermore, we performed the Cochran-Armitage trend test using R software to test for any trends in proportion across different groups. A *p*-value < 0.05 was also considered statistically significant. Additionally, we conducted the Wilcoxon rank sum test using R software to compare the differences in BMI values between groups, with a *p*-value < 0.05 being deemed statistically significant.

### 2.3 Sample preparation

Under sterile conditions, approximately 50 mg of villous tissue from miscarriages or muscle tissue from formed fetuses was collected. The collected tissue samples were then washed thoroughly with sterile physiological saline. The tissue samples can be stored in sterile physiological saline. It is important to ensure that the tissue samples are fully submerged in the preserving solution. Additionally, peripheral blood samples were obtained from the mothers of these fetuses. The samples should then be refrigerated at temperatures between 2 to 8°C during transportation, making sure that the transportation period does not exceed three days. DNA extraction from the tissue and blood samples was performed using the QIAGEN DNeasy Blood & Tissue Kit following the manufacturer’s instructions. The concentration of the extracted DNA was diluted to a range of 20-50 ng/μl for further experimental procedures.

### 2.4 Short tandem repeat analysis

We amplified 17 autosomal Short Tandem Repeat (STR) loci using fluorescently labeled multiplex PCR technique, then employed capillary electrophoresis on an ABI 3500 xL sequencer to detect the alleles of each locus, and imported the resultant electropherogram data into GeneMapper 6.0 software for data analysis and interpretation of the results. Based on the analysis outcomes generated by the GeneMapper 6.0 software, we calculated the peak area ratios of each locus to determine whether the chromosomal ploidy was normal or abnormal (diploid, homozygous uniparental diploid, polyploidy). Additionally, we evaluated maternal contamination by confirming if all of the mother’s polymorphic loci were detected in the fetal sample. Detecting all polymorphic loci indicated the presence of maternal blood contamination.

### 2.5 CNV-seq

We fragmented genomic DNA by random enzymatic digestion and constructed DNA libraries using DNA Library Construction Kit produced by BioelectronSeq Biotechnology Co., Ltd. Subsequently, performed sequencing using the BioelectronSeq 4000 platform at an approximate 1× depth. To determine whether there were any CNVs in the samples, we aligned raw data to the reference genome GRCh37/hg19 and analyzed them for CNVs at a resolution of 100 kb using a Bayesian (33). The results were searched in databases and reviewed in the literature, referring to the DGV database (http://dgv.tcag.ca/dgv/app/home), DECIPHER database (http://decipher.sanger.ac.uk), OMIM database (http://www.omim.org), and following American College of Medical Genetics and Genomics (ACMG) guidelines. We classified CNVs into five categories: pathogenic, likely pathogenic, uncertain significance, likely benign, and benign.

### 2.6 Functional enrichment analysis

We used bedtools software to extract genes from the chromosomal structural abnormality regions detected in abortion tissues based on chromosomal positions. However, considering the detection precision of CNV-seq is 100kb, we excluded genes that were located within 100kb of the edges of the detected regions. Enrichment analysis was performed for functional categories defined in Gene Ontology (GO) and Kyoto Encyclopedia of Genes and Genomes (KEGG). In the current study, we considered the enrichment to be statistically significant when the *p*-value was < 0.05.

## 3 Results

### 3.1 Characteristics of participants

**Figure 1:**
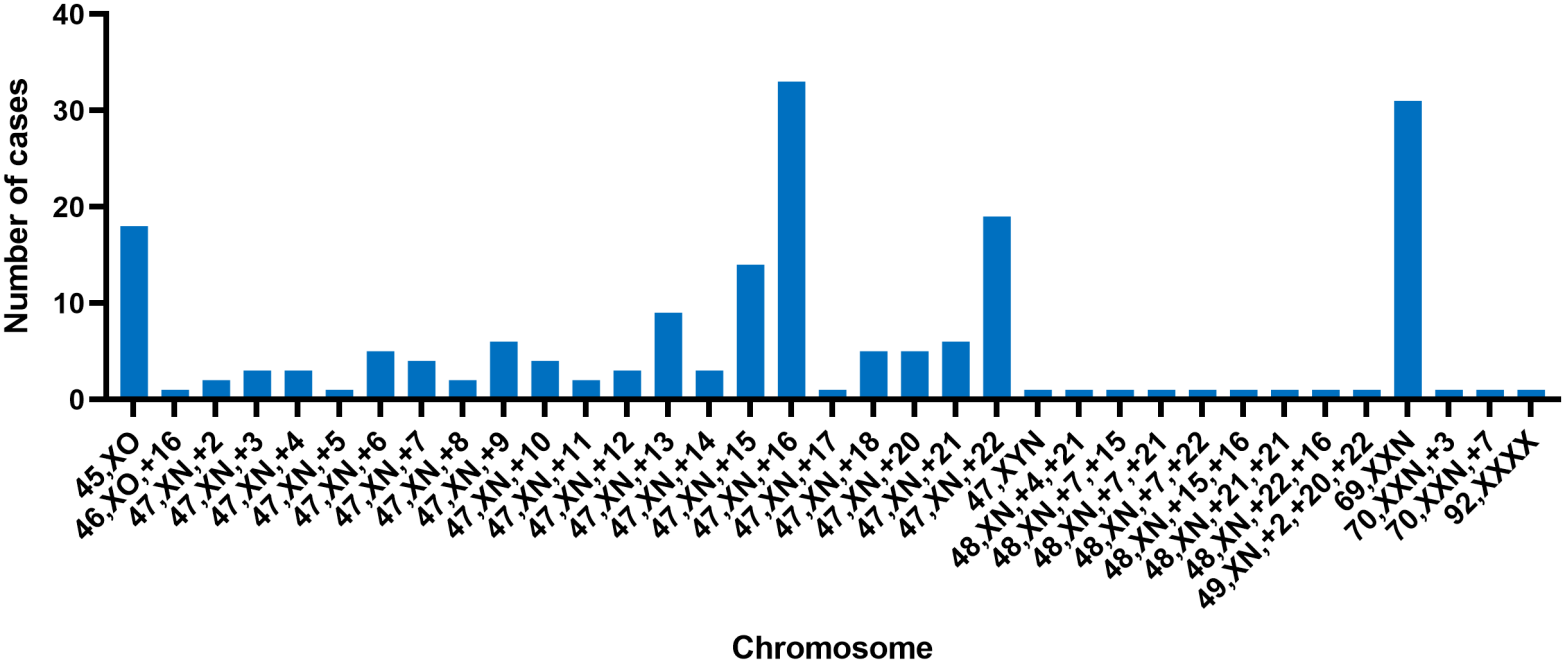
The distribution of numerical chromosomal abnormalities detected on different chromosomes; each bar represents the number of cases with a specific chromosomal abnormality on a given chromosome.

This study involved the collection of a total of 395 fetal samples to undergo CNV-seq testing. Maternal age ranged from 21 to 45 years, with a median of 31 years. Gestational weeks ranged from 4 to 23 weeks, with a median of 10 weeks. Of the pregnant women, 145 (36.71%) were below 29 years, 138 (34.94%) were between 30 and 34 years, 80 (20.25%) were between 35 and 39 years, and 32 (8.1%) were above 40 years in age. The study discovered chromosomal abnormalities in 265 cases (67.09%), while normal karyotypes were observed in the remaining 130 cases (32.91%). Chromosomal abnormalities were composed of 187 cases (47.34%) of numerical abnormalities, 31 cases (7.85%) of structural abnormalities, and 5 (1.27%) cases of both numerical and structural abnormalities (excluding 42 cases of mosaicism). The study detected 138 cases (34.94%) of autosomal trisomy, accounting for 52.08% of the abnormal karyotypes. Among the cases with chromosomal trisomy, chromosome 16 was found to be the most common site, the trisomy of chromosome 16 was observed in 36 cases, accounting for 19.25% (36/187) of all numerical abnormalities, this result is consistent with previous research (30, 31). The second most common trisomy was chromosome 22, which was found in 22 cases, accounting for 11.76% (22/187) of all numerical abnormalities. The study also identified 19 cases of sex chromosome monosomes, accounting for 7.17% of the abnormal karyotypes (**Figure 1**). All except for one case of autosomal monosomy mosaicism on chromosome 21 (karyotype: 45, XN, -21 [15%]/46, XN [85%], mosaicism ratio: 15%), the remaining chromosome monosomy variants were sex-linked. These included 18 cases of 45, XO (one with an additional VUS duplication) and one case of 46, XO, +16. Furthermore, the study discovered 34 cases (8.61%) of chromosome polyploidy, representing 12.83% of the abnormal karyotypes; 19 cases (4.81%) of pCNVs, representing 7.16% of the abnormal karyotypes; 4 cases (1.01%) of likely pCNVs, representing 1.51% of the abnormal karyotypes; and 18 cases (4.56%) of VUS CNVs, representing 6.79% of the abnormal karyotypes (**Table 1**).

**Table 1:** Basic Information of mothers who suffer from miscarriages and overall CNV results of fetuses.

Characteristics	Number	Proportion/constituent ratio (%)
Normal	130	32.91
Numerical abnormalities		
Aneuploidy		
Sex chromosome monosomy	17	4.3
Autosomal trisomy	134	33.92
Sex chromosome trisomy	1	0.25
Autosomal trisomy & Sex chromosome monosomy	1	0.25
Autosomal tetrasomy	1	0.25
Polyploidy		
Hypertriploid	2	0.51
Tetraploid	1	0.25
Triploid	30	7.59
Mosaicisms		
Sex chromosome monosomy mosaicism	6	1.52
Sex chromosome trisomy mosaicism	1	0.25
Sex chromosome monosomy mosaicism & Autosomal Monosomy mosaicism	1	0.25
Autosomal trisomy mosaicism	30	7.59
Autosomal chromosome monosomy mosaicism & Sex Monosomy mosaicism	2	0.51
Autosomal chromosome monosomy mosaicism & Autosomal monosomy mosaicism	1	0.25
Autosomal trisomy & Sex chromosome trisomy mosaicism	1	0.25
Numerical Abnormalities & Structural Abnormalities		
Sex chromosome monosomy & VUS	1	0.25
Autosomal trisomy & VUS	3	0.76
Triploid & VUS	1	0.25
Structural Abnormalities		
likely pCNV	2	0.51
pCNV	13	3.29
pCNV+likely pCNV	2	0.51
pCNV+VUS CNV	4	1.01
VUS CNV	10	2.53
Patient Basic Information		
Age		
≤29	145	36.71
30~34	138	34.94
35~39	80	20.25
≥40	32	8.1
Gestational Weeks		
<8	78	19.75
≥8, <10	151	38.23
≥10	103	26.08
unknown	63	15.59
Clinical Diagnoses		
Sporadic Abortion	185	46.84
Recurrent Abortion	150	37.97
unknown	60	15.19

CNV, copy number variation; VUS, variants of uncertain significance; pCNV, pathogenic CNV.

### 3.2 Chromosomal abnormality distribution across age groups

**Figure 2:**
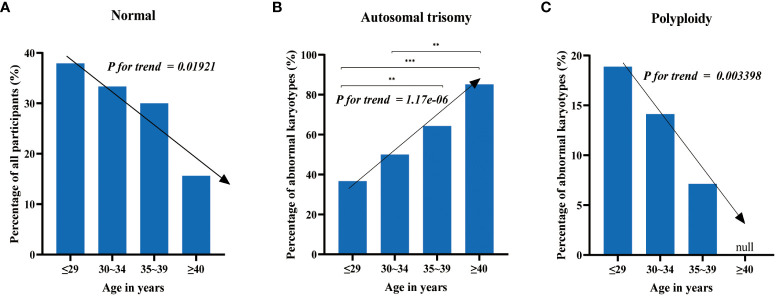
The distribution of chromosomal abnormalities across age groups. **(A)** The detection rate of normal karyotypes decreases with increasing maternal age. **(B)** The proportion of autosomal trisomy in abnormal karyotypes increases with increasing maternal age. **(C)** Chromosomal polyploidy does not occur in women over 40 years old, and the proportion of polyploidy in abnormal karyotypes decreases with increasing maternal age in the other three groups. Test of significance of the chi-square tests: “**” and “***” represent p-value < 0.01 and p-value < 0.001, respectively.

To investigate the association between maternal age and chromosomal abnormalities in cases of spontaneous abortion, participants were categorized into four groups based on maternal age: ≤29 years old, 30-34 years old, 35-39 years old, and ≥40 years old, comprising of 145, 138, 80, and 32 cases, respectively. The samples were subjected to CNV-seq analysis for karyotyping. The detection rates of normal karyotypes for each group were 37.93% (55/145), 33.33% (46/138), 30% (24/80), and 15.63% (5/32), respectively (**Figure 2A**). This indicates a decreasing trend in the detection rate of normal karyotypes with increasing maternal age, with Cochran-Armitage trend test results of z = -2.3414 and *p-*value = 0.01921.

The most common chromosomal abnormalities were autosomal trisomy, accounting for 36.67% (33/90), 50% (46/92), 64.29% (36/56), and 85.19% (23/27) of all chromosomal abnormalities in each group (**Figure 2B**), respectively. This displays an increasing trend of trisomy with increasing maternal age, with Cochran-Armitage trend test results of z = 4.8607 and *p*-value = 1.17e-06. Chromosomal polyploidy, which was less common among chromosomal abnormalities, was not detected in women over 40 years old and accounted for 18.89% (17/90), 14.13% (13/92), and 7.14% (4/56) of all chromosomal abnormalities in the other three groups, respectively (**Figure 2C**), showing a decreasing trend with increasing maternal age, with Cochran-Armitage trend test results of z = -2.9292 and *p*-value = 0.003398. The other chromosomal abnormalities, including mosaicism, monosomy, and structural abnormalities, did not display significant differences among the groups.

### 3.3 Chromosomal abnormality distribution across gestational week

We investigated the detection rates of fetuses with normal karyotypes in early pregnancy (less than 12 weeks) and mid-pregnancy (12 weeks or more) in this study. The rates were found to be 33.33% (103/309) and 43.48% (10/23), respectively, with no significant difference (χ2 = 0.981, *p-*value = 0.322). Moreover, pregnant women were categorized into three groups based on their gestational age: less than 8 weeks (78 cases), no less than 8 weeks, but less than 10 weeks (151 cases), and no less than 10 weeks (103 cases), to examine the association of gestational age with various chromosomal abnormalities in cases of spontaneous abortion. The detection rates of normal karyotypes were 44.87% (35/78), 29.80% (45/151), and 32.04% (33/103), respectively, with no significant difference observed (χ2 = 5.468, *p*-value = 0.065).

**Figure 3:**
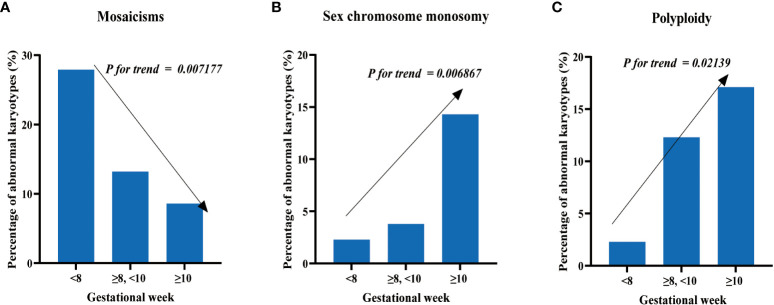
Distribution of chromosomal abnormalities in patients at different gestational weeks. **(A)** The proportion of mosaics among abnormal karyotypes decreases with increasing gestational weeks. **(B)** The proportion of sex chromosome monosomy among abnormal karyotypes increases with increasing gestational weeks. **(C)** The proportion of polyploidy among abnormal karyotypes increases with increasing gestational weeks.

Regarding the proportion of mosaicism among abnormal karyotypes in each group, we found a decreasing trend with increasing gestational age. Specifically, the proportions were 27.91% (12/43), 13.21% (14/106), and 8.57% (6/70) for the three groups, respectively (**Figure 3A**), with Cochran-Armitage trend test results of z = -2.6885 and *p*-value = 0.007177. We also observed an increasing trend in the proportion of sex chromosome monosomy among abnormal karyotypes with increasing gestational age. The proportions were 2.33% (1/43), 12.26% (13/106), and 17.14% (12/70) for the three groups, respectively (**Figure 3B**), with Cochran-Armitage trend test results of z = 2.7032 and *p*-value = 0.006867. In addition, we found an increasing trend in the proportion of chromosomal polyploidy among abnormal karyotypes with increasing gestational age. The proportions were 2.33% (1/43), 3.77% (4/106), and 14.29% (10/70) for the three groups, respectively (**Figure 3C**), with Cochran-Armitage trend test results of z = 2.301 and *p*-value = 0.02139. Nonetheless, the occurrence rates of other chromosomal abnormalities, including trisomy and structural abnormalities, did not display significant differences among the groups.

### 3.4 The association of BMI with sporadic and recurrent abortion

We investigated the association of BMI with karyotype and abortion history in 314 patients, including 145 patients with recurrent abortion and 169 patients with sporadic abortion. Using CNV-seq technology, we divided the patients into two groups: the normal karyotype group and the abnormal karyotype group. Within the abnormal karyotype group, we further categorized them into three subgroups based on different types of chromosomal variations: Numerical abnormalities, Mosaicism, and Structural abnormalities. Within each group, we further divided the patients into a recurrent abortion group and a sporadic abortion group based on their history of miscarriage.

**Figure 4:**
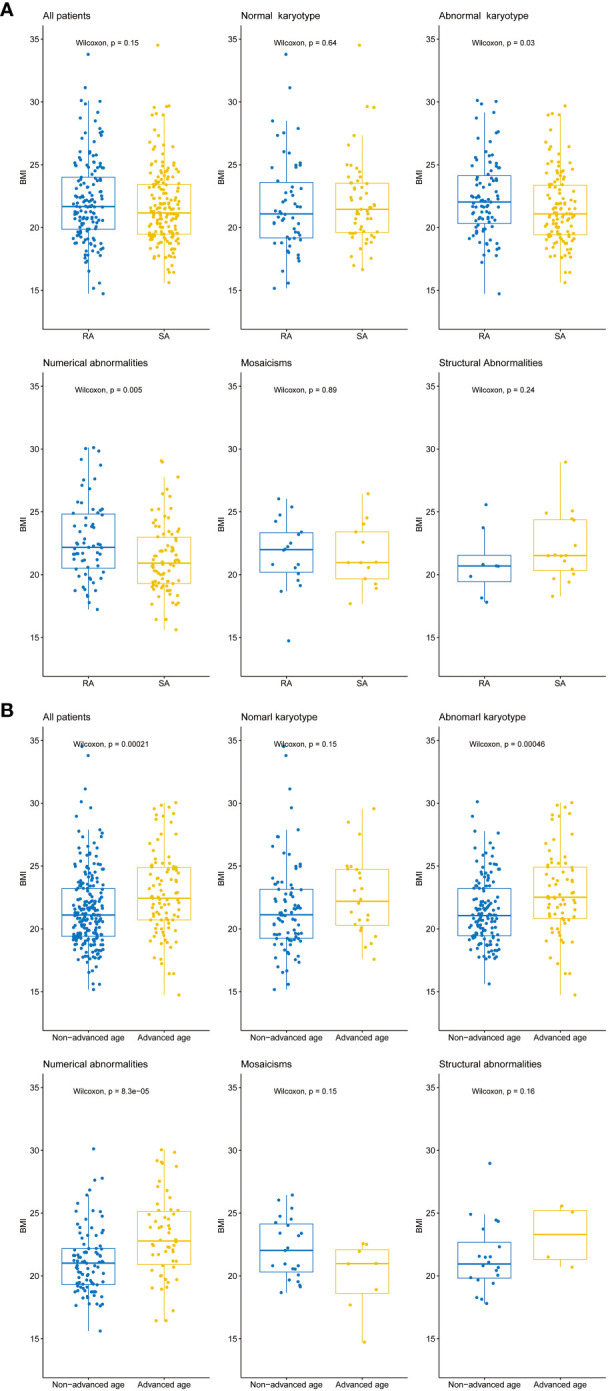
Differences in BMI values between different patient groups. **(A)** Box plots illustrating the distribution of BMI values among all patients, normal karyotype, abnormal karyotype, and different types of abnormal karyotypes (Numerical abnormalities, Mosaicism, and Structural abnormalities). The Wilcoxon rank sum test was performed to evaluate the differences in BMI between patients with recurrent abortion (RA) and sporadic abortion (SA) within each group, *p*-value < 0.05 indicates a statistically significant difference. **(B)** Box plots illustrating the distribution of BMI values among all patients, normal karyotype, abnormal karyotype, and different types of abnormal karyotypes (Numerical abnormalities, Mosaicism, and Structural abnormalities). The Wilcoxon rank sum test was performed to evaluate the differences in BMI between patients with advanced age (35 years and above) and non-advanced age (below 35 years) within each group, *p*-value < 0.05 indicates a statistically significant difference. The horizontal lines within each box indicate the median BMI values, while the upper and lower boundaries of each box represent the upper and lower quartiles, respectively. The whiskers 1.5x interquartile range.

The results showed that among all patients with available BMI data, there was no significant difference in BMI between patients with recurrent abortion and those with sporadic abortion. In the normal karyotype group, there was also no significant difference in BMI between the recurrent abortion group and the sporadic abortion group. However, in the abnormal karyotype group, the recurrent abortion group had a significantly higher BMI than the sporadic abortion group. When analyzing different types of abnormal karyotypes, it was found that among patients with numerical abnormalities, the recurrent abortion group had significantly higher BMI values compared to the sporadic abortion group. There were no significant differences in BMI between the recurrent abortion and sporadic abortion groups in the other two types of abnormal karyotype, mosaicism, and structural abnormalities (**Figure 4A**).

Additionally, we also investigated the relationship between patient age and BMI. The patients were divided into an advanced age group (≥ 35 years old) and a non-advanced age group (<35 years old). The advanced age group had a significantly higher BMI compared to the non-advanced age group. Within the normal karyotype group, there was no significant difference in BMI between the two groups. However, in the abnormal karyotype group, the advanced age group had a significantly higher BMI compared to the non-advanced age group. Among different types of abnormal karyotypes, the advanced age group had significantly higher BMI values compared to the non-advanced age group in patients with numerical abnormalities. However, there were no significant differences in BMI between advanced and non-advanced patients in the other two groups, mosaicism and structural abnormalities (**Figure 4B**).

### 3.5 Comparing pathogenic genes and pathways of unexplained spontaneous abortion in women aged 35 and above and below 35

**Figure 5:**
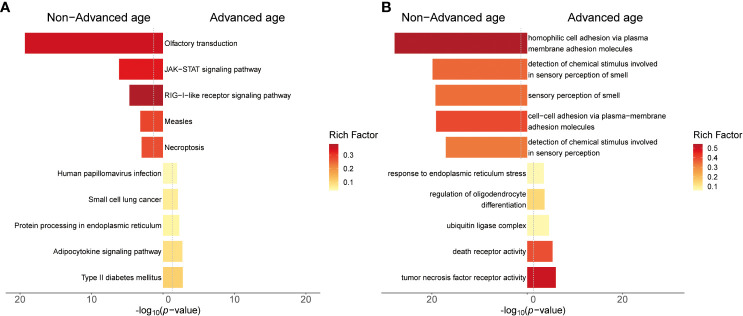
Results of gene enrichment analysis for chromosomal structural variation regions in advanced age and non-advanced age women. **(A)** The top 5 KEGG enrichment pathway of both groups of patients. **(B)** The top 5 GO enrichment terms of both groups of patients. The y-axis represents the KEGG pathway or GO term. The x-axis represents the enrichment significance (-log10 *p*-value). The color of the bars indicates the rich factor, which represents the ratio of the number of target genes to the number of all genes in this pathway or GO term.

We extracted genes from the chromosomal structural abnormality regions detected in the abortion tissues of the non-advanced age group (≥ 35 years old) and advanced age group (<35 years old). We then performed KEGG enrichment analysis (**Figure 5A**) and GO enrichment analysis (**Figure 5B**) to compare their biological functions and pathways. In the chromosomal structural abnormality regions detected in the abortion tissues of the advanced age group, there were 460 genes, of which 164 genes had KEGG annotations and 381genes had GO annotations. In contrast, there were 4499 genes in the chromosomal structural abnormality regions detected in the abortion tissues of those in the non-advanced age group, of which 1465 genes had KEGG annotations and 3125 genes had GO annotations.

The results of KEGG enrichment analysis revealed that in the chromosomal structural abnormality regions detected in the abortion tissues of the advanced age group, the top five enriched pathways were Type II diabetes mellitus, adipocytokine signaling pathway, protein processing in endoplasmic reticulum, small cell lung cancer, and Human papillomavirus infection. In contrast, in the non-advanced age group, the top five enriched pathways for genes within the chromosomal structural abnormality regions detected in abortion tissues were olfactory transduction, JAK-STAT signaling pathway, RIG-I-like receptor signaling pathway, measles, and Necroptosis.

The GO enrichment analysis revealed that in the chromosomal structural abnormality regions detected in abortion tissues of the advanced age group, the top five enriched GO terms for the included genes were tumor necrosis factor receptor activity, death receptor activity, serine-type endopeptidase complex, regulation of oligodendrocyte differentiation, and ubiquitin ligase complex. On the other hand, in the non-advanced age group abortion tissues, the top five enriched GO terms for the included genes within the chromosomal structural abnormality regions were homophilic cell adhesion via plasma membrane adhesion molecules, detection of chemical stimulus involved in sensory perception of smell, sensory perception of smell, cell-cell adhesion via plasma-membrane adhesion molecule and detection of chemical stimulus involved in sensory perception.

## 4 Discussion

Despite the remarkable advances in modern medicine, unexplained miscarriages continue to present a significant challenge for numerous patients and their families. Miscarriages can be caused by a variety of complex factors, such as endocrine imbalances, reproductive tract deformities, infections, immune system complications, genetic abnormalities, and other unidentified causes. Genetic factors are one of the primary causes of miscarriage, and fetal chromosomal abnormalities are the most common genetic factor contributing to miscarriage. This study provides important insights into fetal chromosomal abnormalities and their relationship with maternal age and gestational weeks. Abnormal karyotypes were detected in the majority of cases (67.09%), while normal karyotypes were relatively rare (32.91%). The chromosomal trisomy was most often detected in chromosome 16, this is consistent with prior studies (30). Chromosome 16 is gene-rich but unstable due to a 10% segmental copy sequence, making it prone to frequent non-allelic homologous recombination and gene rearrangements (34). Advanced maternal age is a widely recognized risk that increases the risk of chromosomal abnormalities and spontaneous miscarriage, with the incidence of chromosomal aneuploidy being higher in women over 35 years old (35–38). In our study, we found that the detection frequency of normal karyotypes decreased significantly with increasing maternal age, with the most noticeable difference observed in patients over 40 years old (**Figure 2A**). The rate of normal karyotypes in pregnant women over 40 was only 15.63%, which was almost half the rate of patients aged between 35-39 years old (30.00%). However, the detection rate of normal karyotypes in pregnant women under 40 years was significantly higher than that in pregnant women above 40 years old (χ2 = 4.713, *p*-value = 0.03), which may be attributed to the fact that the autosomal trisomy accounts for a higher proportion of abnormal karyotypes as maternal age increases, but not all chromosomal abnormalities account for a higher proportion of abnormal karyotypes as age increases. In our study results, polyploidy accounts for a lower proportion of abnormal karyotypes as maternal age increases. Therefore, our study suggests that maternal age is an important risk factor for fetal chromosomal abnormalities, especially for autosomal trisomy. However, various chromosomal abnormalities, such as polyploidy, may have different associations with maternal age (39). Previous studies have suggested that the incidence of triploidy is not significantly associated with maternal age (37, 40), yet our findings suggested that the proportion of polyploidy in abnormal karyotypes decreases with increasing maternal age. Therefore, it is recommended to individualize prenatal screening and diagnosis instead of relying solely on maternal age.

In this study, we also explore the correlation between gestational weeks and fetal chromosomal abnormalities. Previous research has suggested that the incidence of chromosomal abnormalities in fetuses is higher during early pregnancy than in the mid-pregnancy phase (30), and triploidy occurs more frequently in pregnancies of less than 11 weeks gestational age (41). However, we did observe no significant difference in the detection rates of fetuses with normal karyotypes in different gestational ages, and the proportion of mosaicisms in abnormal karyotypes decreased with increasing gestational age, while the proportion of sex chromosome monosomy and polyploidy in abnormal karyotypes increased with gestational week. These findings suggest that different types of chromosomal abnormalities may have distinct selection mechanisms during early embryonic development. For example, mosaicisms may be more likely to be eliminated by natural selection in early pregnancy due to their higher level of harmfulness, while sex chromosome monosomy and chromosome polyploidy may be relatively mild. However, more studies with larger sample sizes and longer follow-up periods are required to further support these hypotheses.

The association between BMI and recurrent miscarriage in patients with chromosomal anomalies was a significant finding in our study. There was a significant difference in BMI values between the recurrent miscarriage group and the sporadic miscarriage group in patients with chromosomal anomalies, indicating a potential link between higher BMI values and chromosomal anomalies causing recurrent miscarriage. This finding supports previous research that obesity can negatively affect pregnancy outcomes and result in adverse fetal outcomes (42, 43). However, the BMI values for patients with normal karyotypes and different histories of miscarriage did not differ significantly, suggesting a higher correlation between obesity and recurrent miscarriage caused by chromosomal anomalies. Overall, our study emphasizes the complex relationship between BMI, chromosomal anomalies, and recurrent miscarriage. The findings suggest that BMI should be considered as a potential risk factor for the clinical management of patients with recurrent abortions caused by abnormal karyotypes.

It is generally believed that BMI is influenced by age, with an increase in age resulting in a natural increase in body fat and a decrease in muscle mass (44). Our findings are consistent with this notion. Moreover, we observed a significant difference in BMI values between the advanced age group and the non-advanced age group among participants within the abnormal karyotype. However, no significant difference in BMI was found between these two age groups among participants with normal karyotypes. Based on the results of enrichment analysis, we found that advanced age pregnant women might have different pathogenic genes and pathways compared to the non-advanced age pregnant women who experienced unexplained miscarriages. In advanced age pregnant women, we observed enriched pathways related to metabolic dysregulation and cancer risk. The protein processing in endoplasmic reticulum, pathway involves protein synthesis, modification, and folding processes within cells. Dysfunctional protein processing can lead to protein accumulation and abnormal folding, triggering endoplasmic reticulum stress (45). Endoplasmic reticulum stress is associated with adipocyte dysfunction in obesity and metabolic disorders, and changes in adipokine levels can cause insulin resistance, leading to the development of Type II diabetes mellitus (46, 47). Based on the results of the comprehensive enrichment analysis and BMI analysis, it can be concluded that metabolic dysregulation and obesity have a greater impact on miscarriage in advanced age pregnant women.

Additionally, age is a significant risk factor for the malignant tumors occurrence. While the association between HPV infection and miscarriage risk is debated, HPV infection has been confirmed as a major cause of cervical cancer and other malignancies (48, 49), and chromosomal structural variations are closely related to tumorigenesis (50, 51).

In contrast, the pathogenic mechanisms of miscarriage in non-advanced age pregnant women are more complex and diverse. We observed enriched pathways related to organ development, neural transmission, and immune function. For example, the olfactory transduction pathway may be related to neural system development. homophilic cell adhesion via plasma membrane adhesion molecules and cell-cell adhesion via plasma-membrane adhesion molecule are involved in cell adhesion. cell adhesion is the process of adjacent cells adhering to each other through specific adhesion molecules on the cell membrane. This adhesion maintains the integrity of tissue architecture, facilitates interactions and communication between cells, and participates in various biological processes such as development, tissue repair, and immune response (52, 53). Additionally, the JAK-STAT signaling pathway is an important immunoregulatory pathway involved in processes such as cell growth and immune response (54). Furthermore, the RIG-I-like receptor signaling pathway is a crucial mechanism for recognizing viral infections and triggering immune reactions (55). Necroptosis pathways may induce active inflammatory immune responses (56).

It is important to note that these findings are preliminary and further research is needed to validate and explore these issues in more depth. The genetic and molecular aspects are just part of the complex causes of miscarriage, and other factors such as environmental factors, lifestyle, and genetic backgrounds may also play important roles.

Nevertheless, our study results provide valuable clues for understanding the genetic causes of miscarriage in different age groups. These findings can guide improvements in the quality of care for older pregnant women and other pregnant women. We recommend individualized interventions and enhanced monitoring of metabolic dysregulation, cancer risk, and immune function issues for pregnant women of different age groups in clinical practice. Moreover, our study provides a foundation and direction for future in-depth exploration and comprehensive research in related fields.

In conclusion, our study provides valuable insights into the prevalence and distribution of fetal chromosomal abnormalities using CNV-seq. It also demonstrates the association of maternal age, gestational weeks, and BMI with fetal karyotype abnormalities. These findings have implications for the genetic etiology and risk evaluation of miscarriage.

However, our study also has some limitations. It is crucial to expand our analysis to larger cohorts to obtain a more comprehensive understanding of these associations. Additionally, conducting fundamental experiments will be essential to delve deeper into the underlying molecular mechanisms involved in this phenomenon.

## Data availability statement

The data presented in the study are deposited in the CNGB Sequence Archive (CNSA) of the China National GeneBank Database (CNGBdb) repository, accession number CNP0004438.

## Ethics statement

The studies involving humans were approved by Wenzhou Hospital of Traditional Chinese Medicine Ethics Committee. The studies were conducted in accordance with the local legislation and institutional requirements. The participants provided their written informed consent to participate in this study.

## Author contributions

Conception and design: WC, QZ. Study development and methods: WB, ZL. Medical and technical support: WC, WB, ZL. Collection and assembly of data: XS, LZ. Data analysis and interpretation: WB, QZ. Manuscript writing: WB, QZ. All authors contributed to the article and approved the submitted version.

## Supplementary material

SUPPLEMENTARY TABLE S1 Summarized results.

SUPPLEMENTARY TABLE S2 Gene list in chromosomal structural abnormality regions.
